# Adequate IVIG dosing is associated with an improved long-term outcome in secondary immunodeficiency: A prospective, non-interventional study 

**DOI:** 10.5414/CP204595

**Published:** 2024-07-30

**Authors:** Artur Bauhofer, Ümniye Balaban, Sonja Schimo, Monika Mayer, Jörg Schüttrumpf, Stephan Borte

**Affiliations:** 1 Biotest AG, Dreieich, and; 2 Immune Defect Center, Clinic St. Georg, Leipzig, Germany

**Keywords:** intravenous immunoglobulin (IVIG), immunoglobulin replacement therapy (IgRT), IVIG dosing, secondary immunodeficiency (SID), non-interventional study (NIS), infection rate, quality of life

## Abstract

Objective: To assess the safety, tolerability, and effectiveness of the intravenous immunoglobulin (IVIG) Intratect 50 g/L in immunoglobulin replacement therapy (IgRT) in a prospective, large-scale non-interventional study (NIS). The analysis focused upon patients with secondary immunodeficiency (SID), the most frequent indication for IgRT in this NIS. Materials and methods: Patients were enrolled at 123 centers in Germany. Each patient received IVIG as prescribed by the physician, guided by the Summary of Product Characteristics. Data were acquired from medical records and patients’ questionnaires. Results: In the NIS, 3,563 patients were documented. The main indication for IgRT was SID (73.2%), followed by primary immunodeficiency (14.7%), immune thrombocytopenia (5.8%), and other indications (6.2%). Among the SID patients, 52.9% were male, mean age was 66.5 years, and most (63.8%) were IVIG-naïve. Their annual infection rate improved from 3.7 before documentation in the NIS to 1.1 during the first year of the study. IgG trough plasma levels increased during treatment (> 6 g/L: 44.5% of SID patients at study entry and 64.8% in long-term treatment) and were associated with a trend toward reduced infection rate (p = 0.08). A 1-year infection analysis showed a significantly lower infection risk in the medium- and high-dose groups than in the low-dose group (p = 0.028 and p = 0.017, respectively). Patients’ treatment satisfaction and quality of life improved from baseline. Adverse drug reactions (ADRs) in SID occurred at a low frequency with 0.8% at infusion level. On the patient level, ADRs occurred in 251 (15.3%) SID patients, with chills (7.4%) and pyrexia (0.9%) reported most frequently. Conclusion: Effectiveness, safety, and quality of life confirmed the positive benefit–risk profile of IgRT. Higher IVIG dosages per body weight led to higher IgG plasma trough levels, in turn leading to reduced infection rates. Obese patients may need body-weight-adjusted treatment to reduce the risk of infection.

**What is known about this subject**

- Immunodeficiencies are associated with an increased risk of infection. The most common form is secondary immunodeficiency (SID), which is associated with reduced levels of immunoglobulin G (IgG).
- SID can be induced by conditions such as malnutrition, viral infection, malaria, neutropenia, transplantation, hematological disorder, cancer or side effects of chemo- or radiotherapy, or biologicals such as rituximab.
- Patients with SID and secondary antibody deficiency benefit from immunoglobulin replacement therapy (IgRT), which provides protection against infections and compensates to a certain extent for the underlying immunodeficiency. The optimum trough level is unknown and is dependent on individual needs and the severity of the immunodeficiency.

**What this study adds**

- In this non-interventional study (NIS) with 3,563 participants, IgRT with a specific IVIG of a concentration of 50 g/L, was associated with an increase in IgG trough levels, which were correlated with a reduced incidence of infection. Inadequate dosing, i.e., dose reduction in obese patients, was detected as cause for increased infection under real-world conditions.
- During treatment, the patients’ quality of life and treatment satisfaction improved.
- The number of adverse drug reactions was small, and no new safety signals were detected. The long-term treatment was well tolerated, confirming its positive benefit–risk profile.

## Introduction 

Immunodeficiencies are classified as primary (PID; caused by genetic or other intrinsic factors) and secondary (SID; with external and often multifactorial causes). Factors leading to SID, which is much more common, include malnutrition, human immunodeficiency virus infection, malaria, neutropenia, transplantation, hematological diseases, and side effects of certain medications, in particular those that target B cells [1]. Such treatments are administered across an increasingly broad disease spectrum, and the risk of immunodeficiency is therefore relevant for clinicians in both primary and secondary care [2]. SID has been estimated to be far more common than PID, but unlike in PID, immune function may be recovered if the underlying cause is resolved [3]. 

Hypogammaglobulinemia is also often intrinsic to the pathophysiology of B-cell malignancies, including chronic lymphocytic leukemia (CLL), multiple myeloma (MM), and non-Hodgkin’s lymphoma (NHL), putting patients at a risk of impaired immune function or suppression from both the disease itself and the required treatments. Infections are a leading cause of death in many conditions associated with SID. In CLL, ~ 25 – 60% of deaths have been estimated to be infection-related, while in MM 22% of deaths within the first year of follow-up were infection-related [4]. Infections are usually treated with antibiotics, but these are not always sufficient to manage adequately both infection-related complications and the underlying immunodeficiency. Immunoglobulin replacement therapy (IgRT) is an established intervention for a wide range of symptomatic antibody deficiencies [5, 6]. Today, IgRT is an important option for treating patients who are at risk of SID-related infection, primarily to mitigate their hypogammaglobulinemia and thus to reduce their risk of infection [4, 7]. Intravenous immunoglobulins (IVIGs) consist of polyspecific human IgGs derived from pooled plasma from > 1,000 donations; they thus contain a wide diversity of antibodies and can increase a patient’s circulating IgG levels to improve protection against infection [8]. 

A review by Monleon Bonet et al. [9] pointed out several beneficial effects of IgRT on clinical outcomes and quality of life in SID patients. The review concluded that “disparate definitions, infrequent reporting of statistical significance, and scarcity of clinical trial data after the 1990s present areas for further investigation”. There is therefore still a need for more concrete data to establish a well-founded assessment of the benefits of IgRT in SID [9] and to develop strategies for management of SID [10]. 

The IVIG investigated is a ready-to-use, sugar-free, glycine stabilized preparation. One mL contains normal human immunoglobulin 50 mg with a purity of at least 96% IgG. The distribution of IgG subclasses is IgG1 57%, IgG2 37%, IgG3 3%, and IgG4 3% [11]. Licensed indications include PID syndromes with impaired antibody production, SID with recurrent infections, ineffective antimicrobial treatment, and either proven specific antibody failure or a serum IgG level below 4 g/L. In the EU guideline, recommendations for IgRT changed during the NIS: previously (before January 2019) only those patients who had a high risk of immunodeficiency due to their underlying haemato-oncological disease (CLL, MM, HIV) were eligible to receive IgRT, whereas now, because of changed therapy landscapes with more potent and, correspondingly, stronger immunosuppressive therapies in cancer and other diseases, IgRT is now recommended in all SID patients with pathological susceptibility to infection and a relevant quantitative and/or qualitative antibody deficiency. In order to achieve optimum infection control, a dosage of 0.2 – 0.4 m/kg every 3 – 4 weeks is recommended. While there is no universally accepted IgG threshold to maintain SID patients above to protect against infections in PID patients, a minimum IgG trough level of 6 g/L is recommended [12]. 

This non-interventional study (NIS) (ENCePP register no. EU PAS 7969) was initiated once marketing authorization had been received, with the aim of confirming and further investigating the effectiveness, safety, and tolerability of IgRT in the prevention and treatment of infections in PID and SID under real-life conditions, and at the same time of assessing its effect on patients’ quality of life (QoL). Randomized controlled trials are considered the studies with the highest level of evidence, but they also have limitations [13]. Typically, they have a smaller sample size, and the patient population does not represent the real world situation, as the patient population is highly restricted by in- and exclusion criteria. Here, a non-interventional, observational study design, with a broad population of patients and a large sample size receiving long-term IgRT treatment, was chosen to continue pharmacovigilance surveillance. Results of an interim analysis have been published [14]. In the present paper results of the final analysis are presented. 

Because SID predominates among the various types of immunodeficiencies in our NIS, this indication is discussed in more detail in this article. 

## Materials and methods 

For this study, information was collected from 123 specialist practices and outpatient clinics throughout Germany. The design was prospective, but it incorporated the retrospective inclusion of data from previously treated patients. Before study start, ethics approval was obtained, and the relevant authorities in Germany (the Paul Ehrlich Institute, the National Association of Physicians, and the Statutory Health Insurance Association) were notified. Study design followed the guidance from ENCePP Guide on Methodological Standards in Pharmacoepidemiology and its subsequent releases [15]. 

Data acquisition initially used paper reporting (November 2004 to October 2010) with a transition to electronic case reporting (eCRF) (January 2009 to data cut-off in October 2021); the eCRF reporting was more detailed, so some analyses were conducted for these patients only. The target number of patients was experienced-based, without any test hypotheses, and was ~ 2,500 patients with acquisition by eCRF. 

The studies’ primary objective was to gain additional knowledge on safety and effectiveness of IgRT with Intratect 50 g/L by collecting large-scale data under real-life conditions in patients with a wide range of antibody deficiency syndromes and immune diseases, with different medical histories and pre-treatments, who obtained different dosages of IVIG therapy in different indications. Patients were to be treated in accordance with the prescribing information (SmPC) [11]. Only patients who were able to give informed consent were included in the study. There were no further exclusion criteria. 

Safety variables included adverse events (AEs); temporally associated AEs (≤ 72 hours after the start of infusion) stratified by disease group; AEs of special interest (AESIs); serious adverse events (SAEs); and (serious) adverse drug reactions (ADRs). These were classified by MedDRA System Organ Class (SOC), preferred term and intensity. For effectiveness, variables with regards to SID were as follows: numbers and severity of infections, IgG trough levels and changes from baseline, the Treatment Satisfaction Questionnaire for Medication (TSQM), and global QoL as assessed by patients on a visual analogue scale (VAS). 

Continuous and categorical variables were analyzed by standard descriptive methods. The comparison of patients with IgG < 6 and ≥ 6 g/L for the development of an infection within one year was performed using a multivariate Cox proportional hazard model. Variables included in the Cox model in addition to IgG were patient’s age, weight, and dosage level. Hazard ratios were calculated where relevant. A two-sided p-value of < 0.05 was considered statistically significant. Statistical analysis was performed with SAS (version 9.4, SAS Institute, Cary, NC, USA) and R (R Foundation for Statistical Computing, Vienna, Austria). The R package “survival” was used for calculating Cox regression analysis. 

## Results 

### Patient population 

**Figure 1:**
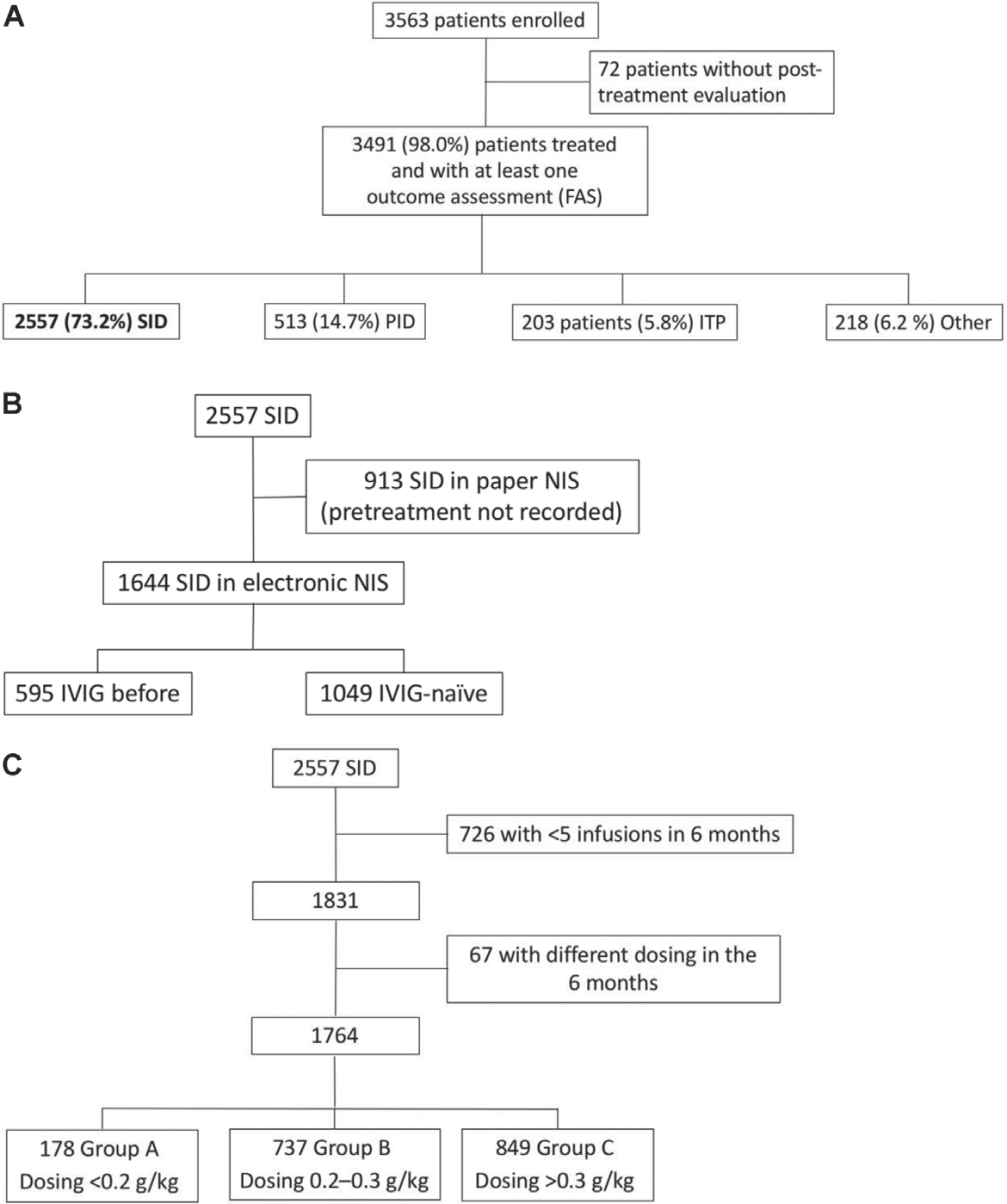
A: Patient enrolment and distribution in the NIS. B: Patient enrolment to the SID subgroups with and without previous IgRT. C: Patient enrolment to the SID subgroups and distribution of dosing per body weight. ITP = immune thrombocytopenia; PID = primary immunodeficiency; SID = secondary immunodeficiency.

A total of 3,563 patients were documented from March 2004 to October 2021. Of these, 3,491 (98.0%) underwent at least one outcome assessment. A breakdown of this population by etiology of immunodeficiency is shown in Figure 1A: ~ 3/4 of these study patients (N = 2,557, 73%) had SID. 

Demographic analysis of the study population did not lead to striking results, except in respect to age: the SID patients were clearly older (mean 66.5 years) than the other etiology groups (PID 53.5 years, immune thrombocytopenia (ITP) 58.4 years, other 45.4 years). This may plausibly be associated with the secondary nature of SID and its main causes. The population was approximately equally divided between the sexes (overall 48.5% male, in the SID patients 52.9% male). 

Of the 3,563 patients, 3,491 (98.0%) received study treatment and had at least one post-treatment evaluation (full analysis set (FAS)), and 2,195 (61.6%) were documented by eCRF, of which 1,644 had SID. 

The main reason for treatment was SID (2,557 patients, 73.2%), followed by PID (513 patients, 14.7%). Fewer patients were diagnosed with ITP (203, 5.8%), and other indications for IVIG were presented by 218 patients (6.2%). The most common “other” indications were multiple sclerosis, repeated bacterial or viral infections, neuropathies, and other autoimmune disorders. 

### Treatment with IVIG 

The ranges of IgRT duration and number of applications were wide, while that of annual numbers of applications was relatively narrow. Between ~ 15% and ~ 37% of the patients had received another IVIG treatment, and 63% were IVIG-naïve. Treatments before NIS start, and with IVIG in the NIS, were very similar in the different study populations; for example, immunoglobulins were used before the NIS in 37.2% of FAS SID patients (Table 1) and in 39.4% of those registered in the electronic NIS (not tabulated). Details of the pre-study and study treatment of the SID patients are shown in Table 1. The duration of documentation per SID patient was substantial, with a median of 403 days. 

### Previous IgRT and dosing subgroups 

The majority of the SID patients were IVIG-naïve at admission to the study (Figure 1B), as exemplified by those whose pretreatment was recorded (after introduction of electronic case reporting): 1,049 (63.8%) were IVIG-naïve, and 595 (36.2%) had previously received IgRT. 

The SID patients who received stable IgRT during the study period (regular infusions: ≥ 5 in 6 months) were further classified according to dose per body weight (Figure 1C): low- (< 0.2 g/kg, group A), medium- (0.2 – 0.3 g/kg, group B,) and high-dose (> 0.3 g/kg, group C). 

Body weight (mean ± standard error of the mean) among the SID patients was highest in the low-dosage group (102.2 ± 1.4 kg), compared with the medium-dosage group (80.8 ± 0.5 kg), and the high-dosage group (65.8 ± 0.5 kg). This trend is to be interpreted as a consequence of physicians’ use of standard IVIG doses without taking account of the patient’s body weight. 

**Figure 2:**
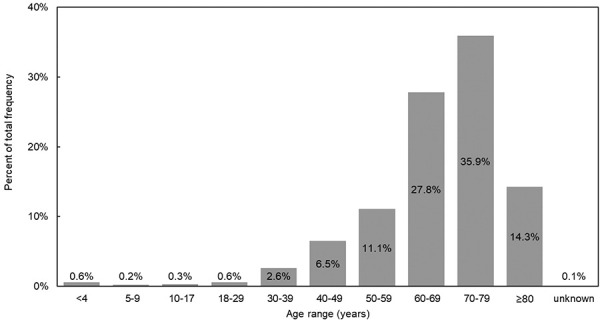
Age distribution of patients with secondary immunodeficiency.

The age distribution of the SID patients is shown in Figure 2. Consistently with their SID etiology, they were relatively old – about 90% were aged 50 years or above. 

### Risk factors 

Physicians were asked to record any pre-existing risk factors for developing IVIG-mediated infusion reactions; a response was obtained for less than half of the 1,644 patients. The most frequently mentioned risk factors (Table 2) were hypertension or vascular disease (64.4%), followed by renal dysfunction (20.8%) and diabetes mellitus (19.6%). 

### IgG trough levels 

Pre-infusion plasma IgG trough levels were lowest before study entry, and they increased steadily during the period of observation. In the SID group, median IgG increased from 5.31 g/L before the first study infusion to 6.65 g/L before infusion 60. The trends in the other etiology groups were similar: e.g., in the PID group there was a corresponding increase from 6.06 g/L to 7.40 g/L. 

**Figure 3:**
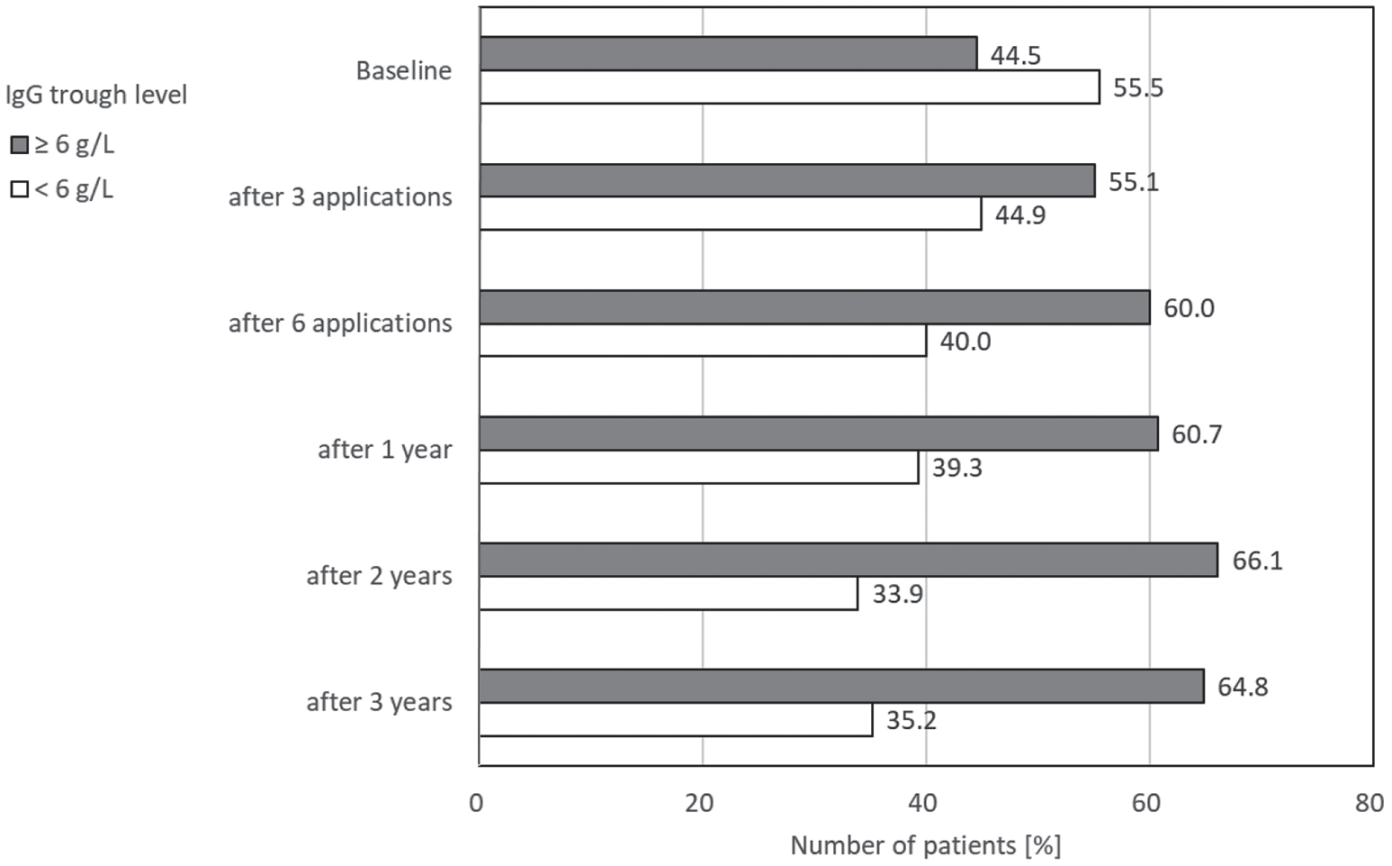
Proportion of patients with secondary immunodeficiency with IgG trough level < 6 g/L and ≥ 6 g/L over time.

To illustrate the changes, the percentages of patients with IgG trough levels above and below 6 g/L were compared for various durations of study treatment. The latter value was chosen on the basis of the EU guideline for PID patients, where a minimum IgG trough level of 6 g/L is recommended. Results are shown in Figure 3: the figure shows the proportion of SID patients with IgG trough level < 6 g/L and ≥ 6 g/L. The proportion of patients below 6 g/L declined from more than one-half of the patients (55.5%) at study start to just over 1/3 (35.2%) after 3 years. 

**Figure 4:**
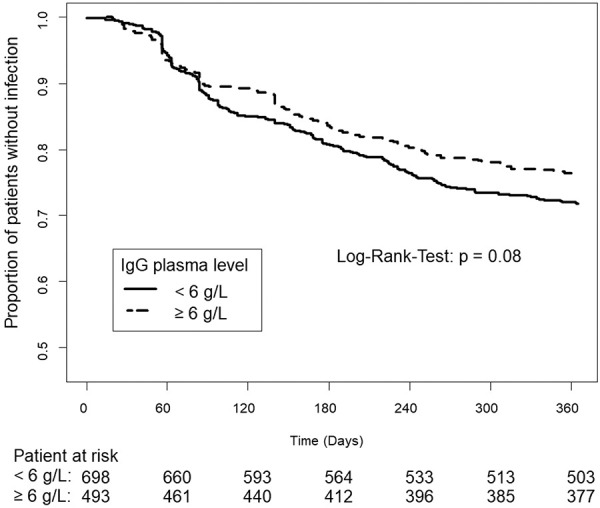
Effect of IgG plasma levels on infections in patients with secondary immunodeficiency within the first year of stable immunglobulin replacement therapy. Kaplan-Meier curves presenting the proportion of patients without infections with IgG plasma levels < 6 g/L and ≥ 6 g/L.

The effect of plasma IgG levels upon the incidence of infections was investigated, and this is summarized in Figure 4. As the figure shows, patients with higher IgG trough levels tended to develop a lower number of infections in the course of time. This approached statistical significance but did not reach it (log-rank test, p = 0.08). 

A Cox regression analysis was performed to identify factors affecting the development of infections within one year. The variables tested were plasma IgG trough level (< 6 or ≥ 6 g/L), patient’s age, patient’s body weight, and dosage group (as defined above). Results are set out in Table 3. The analysis showed that the difference in effect of IgG trough levels alone on infection rate (< 6 g/L or ≥ 6 g/L) did not reach significance (p =  0.155). Age group and body weight likewise did not affect the development of infections. However, treatment with different IVIG per-body-weight dosages had a significant impact on the development of infections. Compared with the low-dose group A (< 0.2 g/kg), the mid- and high-dose groups B and C both showed significantly fewer infections (respectively, p = 0.028 and p = 0.017) in a dose-dependent manner. Thus, patients in the high-dose groups were protected better from infection. 

### Infection episodes 

To investigate the effect of the IgRT on patients’ resistance to infection, the numbers of infection episodes in one year of study participation (annual infection rate (AIR)) were compared with the corresponding numbers in the 3 months before study entry. Results for the SID patients are shown in Table 4. 

The AIR among the SID patients during treatment in the NIS was much lower (1.1) than before NIS entry (3.7). The highest pre-study AIR (4.0) was found for those patients who had not received IgRT before entering the NIS. In both subgroups (with and without previous IgRT), a marked decrease in AIR was documented. Even in the patients with prior IgRT, a lower rate of infections was observed during the NIS; this may be attributed to the inherently more systematic treatment application and data acquisition within the NIS. 

**Figure 5:**
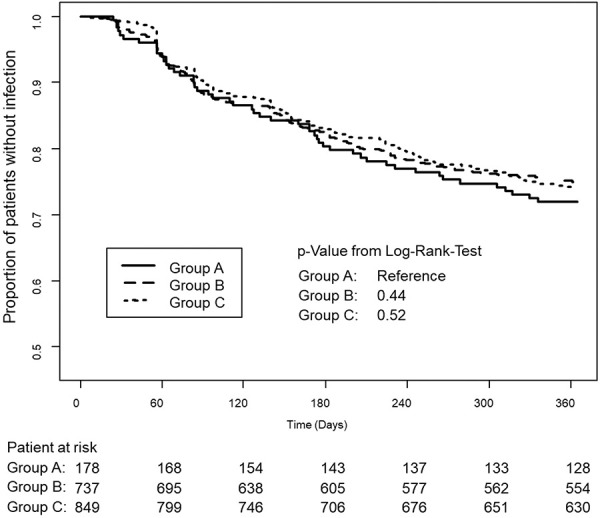
Impact of different IVIG dosing regimens on the occurrence of infections in patients with secondary immunodeficiency. Kaplan-Meier curves presenting the proportion of patients without infections in the low-dose (< 0.2 g/kg, group A), medium-dose (0.2 – 0.3 g/kg, group B), and high-dose group (> 0.3 g/kg, group C).

Kaplan-Meier curves were plotted to investigate the effect of the different per-body-weight dose levels of IVIG during the NIS. These are shown in Figure 5, comparing the 3 dose-level groups A, B, and C (defined above). The proportions of patients with infections over a 360-day period are depicted. The curves for groups B and C showed fewer infections than for group A (lowest dose level), and the curve for group C appeared to be marginally higher than for group B. Thus, a dose-related effect may be present, but it did not reach statistical significance. 

In the first 30 days of treatment, a significantly lower risk of infection in the high-dosage group (C) compared with the low-dosage group (A) was detected by the log-rank test (p = 0.02). However, no differences between the dosage groups were statistically significant at the timepoints 180 and 360 days. Pneumonia was the most common type of infection, documented in 87 (5.3%) of 1,644 SID patients in the NIS, followed by nasopharyngitis in 42 (2.6%) and bronchitis in 30 (1.8%) SID patients. 

### Intake of antibiotics and hospitalization 

Before study entry the AIR for infections requiring antibiotic treatment was 2.7 and for infections requiring hospitalization it was 0.5. In the first year of the NIS, the AIR declined to 0.6 for infections needing antibiotic treatment and for those requiring hospitalization to 0.2 (Table 4). This decline goes in parallel to the physicians’ assessments on antibiotic use. Among a total of 19,742 physicians’ assessments during the NIS, in 1,481 cases (7.5%) a reduction in antibiotic use (including antibiotic prophylaxis) was recorded. 

### Adverse events and adverse drug reactions 

A summary of AEs and suspected ADRs as recorded for the SID patients is shown in Table 5. Adverse events included all events observed, irrespective of causal relationship with the IgRT. In contrast, adverse drug reactions were events considered to have a possible relationship to the IVIG application. 

In total, 2,999 AEs, irrespective of causality, were reported for 723 (32.9%) patients in this NIS. The proportions of patients with AEs were higher in the PID and SID groups (24.4% and 36.9%) than in the ITP and “Other” groups (13.0% and 14.3%). 

AEs temporally associated with the IVIG treatment were documented for 14.6% of all patients. The greatest frequency was in the SID group (16.1%), with 11.0% in the ITP group, 10.8% in the PID group, and 7.1% in the “Other” group. 

Overall, 411 patients (18.7%) experienced 1,427 SAEs. More SAEs (21.9%) were reported in the SID group than in the other groups (PID, 12.1%; “Other”, 5.7%). 

A total of 107 patients (4.9%) died during this NIS. None of these deaths were causally related to the IVIG infusion. All deaths were ascribed to underlying diseases and disease progression (most patients had underlying cancer). Of these deaths, 92 occurred in the SID group. 

The incidence of suspected ADRs relative to the total number of infusions administered was as low as 0.8% for the total study population as well as for the SID subgroup. For the SID group, the incidence of serious ADRs relative to the total number of infusions was 0.07%. 

The severity of all AEs in this NIS was rated as “mild” or “moderate” for the majority of patients (55%). Most of the AEs (64.7%) had resolved or were resolving at the time of reporting. 

The most common AE at preferred-term level, with an incidence ≥ 5%, was “chills”, both in the overall study population (7.4%) and in the SID subgroup (8.3%), independently of whether the patients was previously IVIG-treated or was IVIG-naïve. This was followed by pneumonia and pyrexia (Table 6). Similarly, the most common suspected ADR in the total study population was “chills” (6.5%), again irrespective of previous IVIG treatment. The same was true of the SID subgroup, in which chills were reported as an ADR for 7.4% of the patients, followed by pyrexia (0.9%). The most frequent AESI was “infusion-related reaction”, both in the overall study population (1.3%) and in the SID group (1.5%). 

### Patient satisfaction: Treatment satisfaction questionnaire for medication 

The TSMQ (score 0 – 100, higher scores indicate better satisfaction) is used to assess four domains of treatment satisfaction: effectiveness, side effects, convenience, and global satisfaction. 

The response rates for TSQM questionnaires at baseline were low (less than 20% for the SID patients), and since individual changes from baseline can only be calculated if a patient has both baseline and post-baseline values, only limited data were available to evaluate changes from baseline. 

No median changes from baseline were seen in any of the dose-level groups. Increases in absolute median values for all domains, however, indicated an improvement in satisfaction, starting from 3 infusions and continuing to the last treatment in the total population, for all domains (except for side effects where the median was 100 throughout). For the domain “effectiveness”, the median value improved from a baseline value of 66.7 to 75.0 at the end of treatment. In the same period “convenience” improved from 77.8 to 83.3, and “global satisfaction” improved from 75.0 to 83.3. 

**Figure 6:**
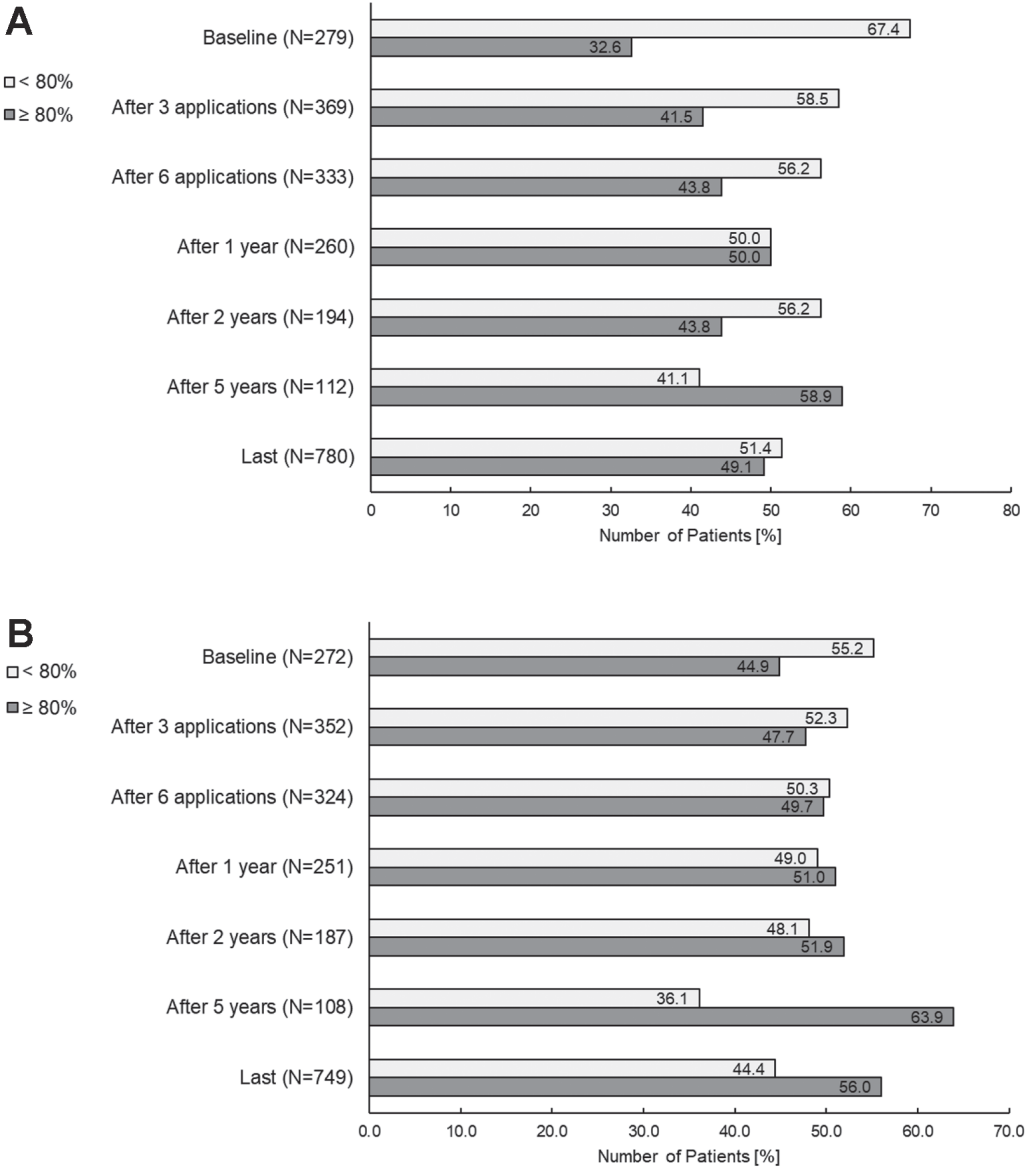
Treatment Satisfaction Questionnaire for Medication. A: Patients with low and high TSQM scores in “effectiveness”. B: Patients with low and high TSQM scores in “global satisfaction”.

Percentages below and above 80 points for the domains “effectiveness” and “global satisfaction” are displayed in Figure 6. An improvement over time was observed in both of these domains for SID patients with high TSQM scores: from 32.6% at baseline to 58.9% after 5 years for effectiveness, and from 44.9% at baseline to 63.9% after 5 years for global satisfaction. 

No relevant differences were observed for the TSQM between the different treatment groups (i.e., with and without previous IgRT) at different timepoints. 

### Patient satisfaction: Quality of life 

General quality of life (Table 7) improved moderately over time independently of whether or not patients had previously received IgRT. Patients with previous IgRT recorded a slightly lower QoL compared with IVIG-naïve patients. 

## Discussion 

This long-term NIS was based upon the observation and analysis of some 3,500 patients being treated under conditions of everyday practice with a specific IVIG over a total study period of more than 17 years. The most frequently encountered indication for receiving IVIG was SID, representing 73% of patients, while PID, ITP, and others made up only ~ 15%, 6%, and 6%, respectively. The relatively high frequency of SID found among candidates for IgRT – compared with PID and ITP – was in accordance with the literature [16]; SID may therefore be regarded as the most important of these indications in clinical practice. For this reason, the present article focusses on the SID patients in the NIS. 

The demographic data for the study population were unremarkable except in respect to age, where the SID patients were – as expected – on average clearly older than those in the other etiology groups. The sexes were approximately equally represented (a potentially important factor owing to sex differences in immune response [17]), both overall and in the SID population. 

Approximately 63% of the SID patients overall were IVIG-naïve; other pre-study treatments included immunosuppressants and corticosteroids. 

The primary goal of IVIG administration is the reduction of infection rate, and indeed this was seen in our study. Specifically, a decrease in AIR for SID patients from 3.7 (2.7 for patients requiring antibiotics, 0.5 for patients with hospitalization) before study treatment to 1.1 (0.6 and 0.2, respectively) after study entry in the NIS was observed. Repeated IgRT demonstrated a benefit in the NIS on the AIR. 

The SID patients receiving stable treatment were classified according to dose per body weight. Body weight was found to be highest (mean 102.2 kg) in the low-dosage group, lower (80.8 kg) in the medium-dosage group, and lowest (65.8 kg) in the high-dosage group. This is interpreted as being due to the use by physicians of standard IVIG doses (1 – 2 complete IVIG bottles; more than 80% of all applications were performed with 200-mL bottles) for at least the majority of patients, irrespective of their body weight. Consistently with this, the occurrence of infections was seen to be lowest in the “high per body weight” dosage group, though without statistical significance. This is consistent with other findings [18, 19]. Overweight patients have a higher risk of developing infection, probably mainly due to underdosing, but the contribution of adverse physiological changes in obese patients cannot exactly be determined. 

The above finding clearly underlines that dosing should be performed in relation to body weight and individual need. A dose reduction for obese patients, as discussed in the literature [20, 21], is inadequate and leads to an increased AIR. An individualized, optimized treatment, with IgG trough-level evaluation, appears mandatory. At present, guidelines for administering IgRT in SID differ among world regions, and patient criteria are often unclear, hampering implementation [4]. These differently weighted criteria include the level of (low) serum IgG, the definition of hypogammaglobulinemia, recurring and/or severe infections, failure of antibiotic treatment, and poor vaccine response. Despite study data indicating beneficial effects of IVIG on morbidity, mortality, and prevention of infection, there is no general accepted guidance for patients who will benefit most from the use of IgRT for infection prevention in SID. 

Adherence to available guidelines correlates with fewer and less severe infections, but for IgRT, adherence is nonetheless generally poor [22]. We observed an increase in the number of patients with IgG trough levels above 6 g/L: 44.5% at baseline rising to 60.0% after 6 administrations and further to 64.8% after 3 years of treatment. The risk of infection was found to be lower in the patients with ≥ 6 g/L IgG, though without statistical significance (p = 0.08), but still 35.2% of the SID patients were below this threshold at year 3. It should be noted that a minimum through level of 6 g/L is recommended for PID patients, which is used here in SID patients as a cut-off value for the analysis. 

In Germany, most institutions do not have an immunologist in charge of taking care of the patients with SID. Closer monitoring of individual patients in co-operation with immunodeficiency centers could improve the patient outcome regarding infections. 

The frequency of suspected ADRs was as low as 0.8% with respect to the number of infusions for both the overall study population and for the SID subgroups and only 0.07% for serious ADRs. None of these serious ADRs led to death. 

The analysis of AEs, temporally associated AEs, AESIs, and ADRs did not show any new or unexpected event pattern that is not already known for IVIG, and hence the safety observed in this NIS is in line with the current label for IVIG. Patients’ treatment satisfaction and their assessment of their global quality of life increased with time from baseline. 

The effectiveness and safety results confirmed the preliminary findings of an earlier interim analysis [14] and of a similar formulation with 100 g/L [23]. Overall, the good safety profile was confirmed, no new signal occurred from this large-scale study. 

The limitations of this study are those generally associated with non-interventional, observational clinical studies, i.e., the flexibility of drug administration and data acquisition, and the limited degree of monitoring and data-cleaning that this allows. A certain degree of discrepancy in the study data is thus inevitable. Not all patients were documented in the extended eCRF, thus some analyses were only possible with a smaller sample size and a less rigid documentation. The data quality in eCRFs is generally better since the electronic system allows for automated queries and more checks. A focus on descriptive data analysis is given. 

## Conclusion 

The results of this NIS of a large, real-world patient cohort confirms the effectiveness of the IVIG investigated in reducing infection episodes. 

The SID study population showed clear positive changes in IgG level during study treatment: At study begin, 44.5% of the patients had an IgG trough level above 6 g/L, and this fraction rose to 60.0% after 6 administrations and further to 64.8% after 3 years of treatment. Consequently, in 7.5% of the patients with SID, the intake of antibiotics could be reduced. The annual infection rate among the SID patients fell from 3.7 before study treatment to 1.1. Patients with higher IgG trough levels (> 6 g/L) tended to develop a lower number of infections, suggesting that SID patients may also benefit from targeted dosing regimens. Compared with the low-dose group (< 0.2 g/kg), the mid- (0.2 – 0.3 g/kg) and high-dose (> 0.3 g/kg) groups showed significantly fewer infections in the multivariate Cox regression analysis, but not in the univariate analysis using the log-rank test in a dose-dependent manner. 

ADRs affected 15.3% of the SID patients, corresponding to a per-infusion incidence of 0.8% and for serious ADRs of 0.07%. No new safety signal was detected. The long-term treatment was well tolerated, supporting the positive benefit–risk profile of the IVIG investigated. 

Results from the Treatment Satisfaction Questionnaire for Medication improved during the study, as did the patients’ self-reported global quality of life over time, but this did not reach statistical significance. 

## Acknowledgment 

We thank Paul Woolley for assistance with writing the manuscript. 

## Authors’ contributions 

A. Bauhofer: Designing the study, supervision of the study, analysis of the study, interpretation of the study results, main author of this publication. Ü. Balaban and S. Schimo: Analysis and interpretation of the study results. M. Mayer, J. Schüttrumpf and S. Borte: Interpretation of the study results. 

## Funding 

The study was funded by Biotest AG, Dreieich, Germany. 

## Conflict of interest 

A. Bauhofer, Ü. Balaban, S. Schimo, M. Mayer, and J. Schüttrumpf are employees of Biotest AG. 

**Table 1.** Treatment of SID patients.

Treatments before NIS start	
Treatment	No. of patients^a^
Immunoglobulins^b^	952 (37.2%)
Immunosuppressants	390 (15.3%)
Corticosteroids	1,005 (39.3%)
Other	512 (20.0%)
None	690 (27.0%)
IVIG treatment^c^	
Treatment duration (days)	403 (157 – 921)
Number of applications per subject	12.0 (6 – 28)
Annual number of applications	12.8 (10.6 – 15)
Dose per kg body weight (g/kg)	0.3 (0.3 – 0.4)
Speed of infusion (mL/kg/h)	2.0 (1.4 – 2.7)
Initial speed of infusion (mL/kg/h)	1.0 (0.9 – 1.3)
Maximum speed of infusion (mL/kg/h)	2.4 (2.0 – 3.0)

^a^Percentages are based on the SID patients who received at least 1 IVIG administration and for whom at least 1 post-administration assessment was available; N = 2,557. Multiple answers possible. ^b^I.e., non-naïve patients (see text). ^c^Medians are shown, with interquartile ranges in square brackets. SID = secondary immunodeficiency; NIS = large-scale non-interventional study.

**Table 2.** Risk factors in patients with secondary immunodeficiency..

Risk factor (multiple answers possible)	N responses	n “yes” (%)
Hypertension or vascular disease	573	369 (64.4%)
Renal dysfunction	557	116 (20.8%)
Diabetes mellitus	561	110 (19.6%)
Thromboembolic events	562	57 (10.1%)
Autoimmune disease	555	42 (7.6%)

Total N = 1,644. The most frequently mentioned risk factors (> 5%) are shown.

**Table 3.** Cox regression analysis of the development of infection within 1 year in patients with secondary immunodeficiency.

	Hazard ratio	95% CI	p-value
IgG trough < 6 g/L	Reference	–	–
IgG trough ≥ 6 g/L	0.84	0.66 – 1.07	0.155
Age group ≤ 6 years	Reference	–	–
Age group > 18 years	1.08	0.82 – 1.43	0.586
Body weight < 60 kg	Reference	–	–
Body weight 60 – 80 kg	0.86	0.63 – 1.16	0.324
Body weight > 80 kg	0.72	0.49 – 1.06	0.098
Dose group A (< 0.2 g/kg)	Reference	–	–
Dose group B (0.2 – 0.3 g/kg)	0.59	0.36 – 0.94	0.028
Dose group C (> 0.3 g/kg)	0.59	0.39 – 0.91	0.017

**Table 4.** Infection episodes before study entry and in 1 year of study participation.

Infections before study entry and mean (AIRs).			
	SID patients	SID patients with IVIG before NIS	SID patients without IVIG before NIS
	(N = 1644)	(N = 595)	(N = 1,049)
Number of infections in the last 3 months^a^ and (AIR)^b^	843 (3.7)	237 (3.0)	606 (4.0)
Number of antibiotic-requiring infections in the last 3 months^a^ and (AIR)^b^	613 (2.7)	160 (3.1)	453 (2.0)
Number of infections requiring hospitalization in the last 3 months^a^ and (AIR)^b^	114 (0.5)	29 (0.4)	85 (0.6)
AIR in the first year of the NIS			
Annual infection rate – all infections	1.1	1.3	1.0
AIR for infections requiring antibiotics^c^	0.6	0.7	0.6
AIR for infections requiring hospitalization	0.2	0.2	0.2

Total N = 1,644. Not all 1,644 patients provided information on whether they had an infection before NIS entry. ^a^The numbers for the last 3 months before study participation relied on the patients’ recollection. ^b^The annual infection rate was calculated on the basis of 3-month values and patients with data available. ^c^Including patients with antibiotic prophylaxis. AIR = annual infection rate; SID = secondary immunodeficiency; NIS = large-scale non-interventional study.

**Table 5.** Adverse events and suspected adverse drug reactions (SID patients).

	All patients		All SID patients		SID patients with IVIG before NIS		SID patients without IVIG before NIS	
	(N = 2,195)		(N = 1,644)		(N = 595)		(N = 1,049)	
No. of IVIG infusions	81,033		58,187		25,205		32,982	
	n_P_	n_E_	n_P_	n_E_	n_P_	n_E_	n_P_	n_E_
Adverse events: any	723 (32.9%)	2,999	607 (36.9%)	2,618	253 (42.5%)	1,158	354 (33.7%)	1,460
Temporally associated^a^	321 (14.6%)	656	264 (16.1%)	522	115 (19.3%)	234	149 (14.2%)	288
Serious	411 (18.7%)	1,427	360 (21.9%)	1,300	151 (25.4%)	561	209 (19.9%)	739
Fatal	107 (4.9%)	190	56 (3.4%)	98	22 (3.7%)	32	34 (3.2%)	66
Adverse drug reactions: any	304 (13.8%)	608	251 (15.3%)	479	105 (17.6%)	201	146 (13.9%)	278
Serious	20 (0.9%)	54	16 (1.0%)	40	4 (0.7%)	14	12 (1.1%)	26
Fatal	–	–	–	–	–	–	–	–

Total N = 2,195 and 1,644 with SID. n_P_ = number of patients (percentage based on N); n_E_ = number of events. ^a^≤ 72 hours after start of infusion. SID = secondary immunodeficiency; NIS = large-scale non-interventional study.

**Table 6.** Most frequent adverse events (incidence ≥ 5%) and adverse drug reactions by MedDRA preferred term.

	Patients in NIS N = 2,195		SID patients N = 1,644	
Preferred term	Adverse events	Adverse drug reactions	Adverse events	Adverse drug reactions
Chills	162 (7.4%)	143 (6.5%)	137 (8.3%)	121 (7.4%)
Pneumonia	97 (4.4%)	–	87 (5.3%)	–
Pyrexia	95 (4.3%)	21 (1.0%)	81 (4.9%)	14 (0.9%)

SID = secondary immunodeficiency; NIS = large-scale non-interventional study.

**Table 7.** General quality of life (full analysis set).

	SID patients (N = 442)	IVIG before NIS (N = 146)	No IVIG before NIS (N = 296)
Baseline	10	9	10
After 3 infusions	10	10	10
After 1 year	11	10	11

Score range 0 – 15, optimum 15; medians are shown. SID = secondary immunodeficiency; NIS = large-scale non-interventional study.
